# Effect of Scapular‐Focused Interventions on Pain and Disability in Neck Pain With Mobility Deficits: A Randomized Controlled Trial

**DOI:** 10.1155/prm/3143187

**Published:** 2026-07-16

**Authors:** Nithin Prakash, Karvannan Harikesavan, Joshua Cleland, Hemant K. Kalyan

**Affiliations:** ^1^ Department of Physiotherapy, Manipal College of Health Professions, Manipal Academy of Higher Education, Manipal, Karnataka, India, manipal.edu; ^2^ Physical Therapy Program, Department of Rehabilitation Sciences, School of Medicine, Tufts University, Boston, Massachusetts, USA, tufts.edu; ^3^ Department of Orthopaedics and Sports Medicine, Manipal Hospital Bangalore, Old Airport Road, Bangalore, India

**Keywords:** acute neck pain, disability, mobility deficits, neck pain, scapula dysfunction

## Abstract

**Background:**

This study aimed to determine the isolated effect of scapular‐focused interventions in individuals with neck and mobility deficits on pain and disability.

**Methods:**

A total of 108 subjects with neck pain with mobility deficits were randomly allocated into two groups using block randomization. The experimental group received scapular‐focused interventions (targeting the lower trapezius, middle trapezius, and serratus anterior), and the control group received neck‐focused interventions (neck strengthening exercises). Both groups received 6 weeks of supervised treatment and 6 weeks of unsupervised treatment. Pain, disability, and pressure pain threshold (PPT) were assessed at baseline, 6 weeks and 12 weeks. A repeated measure generalized linear model with group as the between‐subject factor and time as the within‐subject factor was used for statistical analysis, with post hoc Tukey’s tests used for pairwise comparisons.

**Results:**

Pain and disability significantly improved over time (*p* < 0.001, *η* *p* ^2^ = 0.87–0.95, large effect) in both groups. No significant group × time interaction was observed for either pain (*p* = 0.10) or disability (*p* = 0.154). PPT following the isolated scapular interventions exhibited a greater improvement in PPT at the levator scapulae and upper trapezius compared to the neck exercise group. This was observed at postintervention and follow‐up periods, with a significant group × time interaction (*p* < 0.001). PPT of the spine improved significantly over time in both groups. No significant effect of age or sex was observed on pain or disability.

**Conclusion:**

The findings of the study suggest that isolated scapular‐focused interventions are equally effective in reducing pain and disability and significantly better in improving PPT in individuals with neck pain and mobility deficits compared to neck exercises alone.

**Trial Registration:** Clinical Trials Registry–India: CTRI/2021/10/037543

## 1. Introduction

Neck pain is a “pain perceived as arising in a region bounded superiorly by the superior nuchal line, laterally by the lateral margins of the neck, and inferiorly by an imaginary transverse line through the T1 spinous process” [1]. It is a multifactorial pathology with a prevalence of 3551.1 per 100,000 persons and affects 2/3 of the population at some point in life [2]. Neck pain has been classified into neck pain with mobility deficits, neck pain with movement coordination impairment, neck pain with radiating pain, and neck pain with headache [3]. Neck pain with mobility deficits typically presents as central or unilateral neck pain, with a restricted range of motion and associated shoulder symptoms [4]. Risk factors include demographic (age and gender), psychological/psychosocial factors (emotional problems, low job satisfaction, etc.), ergonomic factors, and mechanical/physical dysfunction (history of neck/back pain and scapular dysfunction) [5, 6].

Scapular dysfunction is an aberrant position and/or movement of the scapula [7]. Studies indicate that individuals with neck pain may have an aberrant scapular movement pattern [8, 9]. The scapula and neck share common muscular attachments, and scapular dysfunction can lead to altered functions of the axioscapular muscles, leading to abnormal cervical spine loading [6]. This irregular loading may reduce the strength of cervicoscapular muscles in individuals with neck pain and mobility deficits. Literature has shown that there is a reduced pain threshold in the neck and scapular muscles, a reduced range of motion, delayed activation, and lowered scapular muscle strength among individuals with neck pain and mobility deficits [10–14].

A recent trend, including treatment of the scapula and scapulothoracic muscles, has been observed in the management of individuals with neck pain. This includes passive and active interventions addressing the scapulothoracic muscles [15–20]. This suggests active strengthening of scapular muscles along with neck exercise is beneficial in the management of neck pain. Also, a systematic review by Seo et al. reported the need for quality evidence to determine the effect of scapular strengthening exercise for pain, disability, and quality of life in individuals with neck pain [21].

This emphasizes the need for an exercise program that addresses the balance in muscle activation to achieve the proper length‐tension relationship. As the scapula serves a crucial biomechanical link between the spine and upper extremity, any alteration in its position can affect the length‐tension relationship of the cervicoscapular muscles. This mainly affects the upper trapezius and levator scapulae, thereby increasing the mechanical load on the cervical spine, leading to pain and disability. The scapular‐focused interventions aim to stabilize the muscular activity around the scapular region and restore the normal scapular position. It is hypothesized that the reduced tension on the cervicoscapular muscles may potentially reduce pain and disability. Despite the available evidence on scapular dysfunction in individuals with neck pain and correction strategies, the effect of scapular‐focused interventions remains insufficiently studied among individuals with neck pain. Hence, this study aimed to determine the isolated effect of scapular‐focused interventions in individuals with neck and mobility deficits on pain and disability.

## 2. Materials and Methods

### 2.1. Participants

This study adhered to the Consolidated Standard of Reporting Trials (CONSORT) 2025 guidelines [22]. Three hundred and sixty‐three subjects were screened for scapular involvement via the scapular assistance test, scapular retraction test, the reduced active rotation range of motion of the neck, increased pain sensitivity in the cervical spine and periscapular muscles, and muscle strength testing of scapular muscles. Following the screening and assessment, informed consent was obtained from the eligible subjects, and baseline data were collected from those agreeing to participate.

Individuals between the ages of 18 and 55 who experienced neck pain with scapular involvement, a numerical pain rating score (NPRS) greater than 3, a neck disability index (NDI) of more than 10%, neck pain lasting less than 38 days, cervical pain, and limited cervical active range of motion and segmental mobility were included in the study [3]. Less than 38 days of symptoms was used as an inclusion criterion based on the fact the Clinical Practice Guidelines on neck pain describe individuals with symptoms less than 38 days to be consistent with the neck pain with mobility deficits subgroup. Participants with cervical myelopathy, neoplastic conditions, upper cervical ligamentous instability, vertebral artery insufficiency, inflammatory or systemic diseases, a history of cerebrovascular headache, radiculopathy symptoms, or recent upper extremity or neck trauma (in the past 6 months), such as a history of whiplash injury, prior neck or shoulder surgery, shoulder impingement or upper quadrant dysfunction, rotator cuff pathology, thoracolumbar fascia tightness, shoulder pain, or neck pain resulting from cardiovascular pathology, were all excluded from the study, as the Clinical Practice Guidelines eliminate these individuals from the neck pain with mobility deficits subgroup.

The study was approved by the institutional research committee of Manipal College of Health Professions, Manipal, and the ethical committee of Manipal Hospitals, Bangalore.

### 2.2. Study Design

A single blind randomized controlled trial.

### 2.3. Participant Recruitment

The participants were recruited from the Physiotherapy‐Out Patient Department, Manipal Hospitals, Bangalore.

### 2.4. Interventions

One hundred and eight participants were enrolled in the study and randomly allocated into the experimental group, receiving scapula‐focused interventions, and a control group, receiving neck‐focused interventions. The subjects in both groups received the randomly assigned intervention for 12 weeks, where the initial 6 weeks were supervised and the following 6 weeks were unsupervised.

### 2.5. Scapula‐Focused Interventions

The scapula‐focused interventions included scapula mobilization followed by gentle range of motion exercises of the neck, shoulder, and scapula. Following the initial warm‐up, the subjects were prescribed specific scapular strengthening exercises. The exercises were directed at the scapula muscles and included prone flexion, prone extension, side‐lying external rotation, side‐lying forward flexion, prone abduction with external rotation, prone unilateral row, diagonal exercise, dynamic hug, bilateral external rotation, bilateral serratus anterior punch, and push‐up plus exercises. The exercises ended with stretching of the upper trapezius and levator scapulae muscles and can be seen in the Supporting Information (available here) [23, 24].

### 2.6. Neck‐Focused Interventions

The interventions in the control group included gentle pain‐free range of motion exercises of the neck in all planes, gentle mobilization to the cervical spine (posterior to anterior mobilization), chin tuck, cervical extension, shoulder shrug, shoulder roll, shoulder retraction, cranio‐cervical flexion and cranio‐cervical extension, cervical extension‐dynamic isometrics, and cervical flexion‐dynamic isometrics and can be seen in the Supporting Information. The neck endurance exercises were added gradually to the previous exercises on the basis of the subject’s performance with the exercises [25–27].

The exercises in both groups were initiated with fewer repetitions and simpler exercises at the beginning. The exercises gradually progressed with increasing the number of repetitions, increasing the number of sets of exercises, and the addition of resistance using resistance bands or weights based on patient’s improvement with the exercises. The exercises progressed with the above parameters if there was no pain or fatigue reported by the subjects.

During the supervised treatment, the subjects in both groups received interventions in a similar pattern. The subjects received supervised intervention sessions for a duration of 30–45 min/week. The exercise load was determined individually, and once the subject was able to complete 3 sets of 15 repetitions of exercise without pain or fatigue, they were progressed to increased resistance. If the subject experienced pain or fatigue while doing the exercise or reported an increase in the pain score by two points, the subsequent session was started with a lower load, a lower repetition or a combination of both. If the subject did not mention any discomfort or pain during or after the exercise, the repetitions and the load gradually increased. The subjects were familiarized with the exercises for 6 weeks. Following the supervised treatment, the subjects were asked to continue exercising at home for an additional 6 weeks. The subjects were provided with exercise charts to ensure the proper form of exercise. The exercise charts included the details of the exercises, including the frequency and number of repetitions and sets. The subjects were asked to follow the pattern of increasing the load gradually along with the repetitions. Adherence and compliance with the exercise were monitored via the Exercise Adherence Rating Scale and the Correctness of Exercise Performance Scale [28, 29].

The contact details of the patients were collected, and follow‐ups were made via text/phone calls. The follow‐up included motivational cues to encourage adherence to the exercises, and positive reinforcements regarding the benefits of exercises were given at certain intervals. Additionally, the subjects were asked to contact the clinic if they had any concerns regarding the exercises or progression of the exercises.

If any pain or fatigue was reported while the participants were exercising, the exercise intensity was lowered, and the repetitions or the weights were noted, and the subject was instructed to continue with a lower weight or repetition. There were no adverse events noted during any phase of the trial, and no severe flares were noted during the trial. A mild increase in pain due to soreness was reported but spontaneously resolved within 48 h.

### 2.7. Outcomes

Outcomes were assessed at baseline, 6 weeks (posttreatment), and 12 weeks (follow‐up). The study included the primary outcomes of pain and disability and the secondary outcome of pressure pain threshold (PPT).

Disability: Disability was assessed via the NDI scale. The scale measures disability from 0–50, where 0 indicates no disability and 50 indicates the highest degree of disability. The final score is obtained by adding the sum score and multiplying by 2 to obtain a percentage of disability. The NDI is a valid and reliable tool used to assess the degree of disability in subjects with neck pain. The NDI has been shown to display excellent reliability (0.88). The minimum detectable change for the NDI is 6.9 points, and the minimum clinically important difference is 5.5 points [30, 31].

Pain: Pain was assessed via the NPRS. The NPRS assesses the intensity of pain, where 0 is considered no pain/pain free and 10 is considered the worst pain imaginable. The patient was asked to report their current level of pain. The NPRS is a widely used scale for assessing pain in individuals with musculoskeletal disorders and has been shown to exhibit a reliability of 0.76. The minimum detectable change for the NPRS is 2.6 points, and the minimum clinically important difference is 1.5 points [31, 32].

PPT: Pressure algometer was used to assess the PPT. The pressure algometer has shown a high reliability of 0.81–0.86 [33]. The minimum detectable change for the PPT using an algometer is 17%–33% [34]. The round tip of the instrument was perpendicularly applied to the painful area, and the reading at which the subject experienced pain was recorded. Following a rest period of 30 s between each measurement, a total of three readings were taken. Testing was performed on the standardized measurement sites for upper trapezius, levator scapula, and cervical spine [4, 35–37]. Assessment of the PPT of the spine was performed centrally over the C5 and C6 spinous processes, and the mean of the two measurements was used for analysis. PPTs for the levator scapulae and upper trapezius were assessed unilaterally on the symptomatic side.

### 2.8. Sample Size

The sample size was calculated based on the standard deviation and minimum clinically important difference of the primary outcomes (NPRS and NDI), study power of 80%, and an alpha level of 0.05. The sample size was obtained via the formula *n* = 2 × 2.82 × SD2/MCID2. SD = 8.46, MCID = 5, *n* = 45 for the NDI and SD = 1.07, and MCID = 4.5 *n* = 45. The outcome measure with the highest sample size was selected, with 45 patients per group, and a dropout rate of 20%, which was calculated via a formula (n/1 rate). The total number of participants in one group, including those who would potentially drop out, was 54.

### 2.9. Blinding and Randomization

The participants were randomly allocated into experimental and control groups with an allocation ratio of 1:1 via block randomization. Eleven blocks of size 10 were used to randomize subjects. Opaque envelopes were used for concealed allocation. The supervisor, who was not involved in subject recruiting, signed the sealed‐opaque envelopes. Following the participant’s report, an impartial adjudicator chose an envelope and informed the treating therapist of the assigned intervention. The outcomes in the study were assessed and collected by an assessor who was an experienced physical therapist who was blinded to the treatment groups.

### 2.10. Statistical Analysis

Statistical analysis was done using Jamovi (Version 2.3.28) [38]. Means, medians, and standard deviations were used to report demographics of the included subjects. The Shapiro–Wilk’s test was used to assess normality, and Levene’s test was used to assess the homogeneity of variance among the included subjects. A repeated measure generalized linear model with group as a between‐subject factor and time as a within‐subject factor was used to determine the differences between the groups, time, and group interactions between subjects. Post hoc analysis for statistically significant outcomes was performed via Tukey’s test. A secondary analysis was performed to determine the effect of the covariates via linear regression.

## 3. Results

**FIGURE 1:**
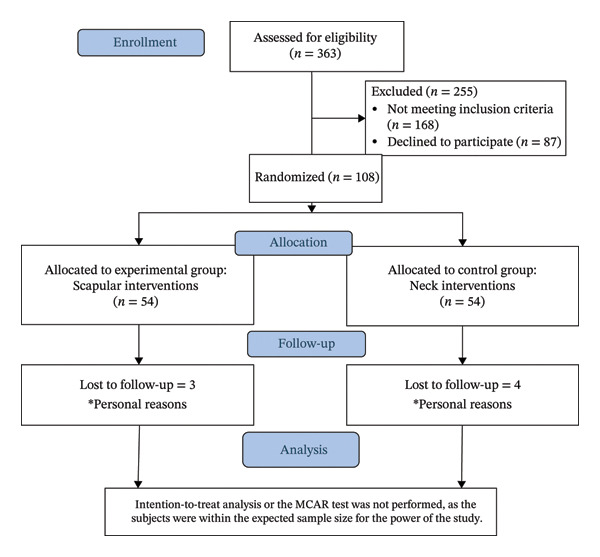
Flow of the study.

A total of 363 subjects were screened, and 108 subjects were included in the study. The flow of the study is depicted in Figure 1.

### 3.1. Participant Characteristics and Baseline Values

Among the 108 subjects, 83 (77%) were females, and 25 were males (23%). The mean age of the included subjects (*n* = 108) was 28.58 ± 5.51 years. The demographics of the included subjects and baseline characteristics of the subjects are provided in Table 1.

**TABLE 1:** Demographic and baseline outcome details of the included participants.

Descriptives	Group	*N*	Mean	SD
Age (in years)	Experimental	54	29.111	5.127
	Control	54	28.056	5.874

Pain	Experimental	54	6.241	1.516
	Control	54	5.852	1.420

Disability	Experimental	54	23.444	5.414
	Control	54	24.222	4.373

PPT Levator Scapulae	Experimental	54	1.963	0.756
	Control	54	2.142	0.828

PPT Upper Trapezius	Experimental	54	2.092	0.454
	Control	54	2.110	0.478

PPT Spine	Experimental	54	0.977	0.498
	Control	54	1.149	0.455

Lower Trapezius Strength (KgF)	Experimental	54	3.074	0.843
	Control	54	4.056	1.235

Middle Trapezius Strength (KgF)	Experimental	54	14.370	1.866
	Control	54	14.685	2.188

Serratus Anterior Strength (KgF)	Experimental	54	15.278	2.375
	Control	54	15.000	1.981

Abbreviations: kgF, kilogram force; PPT, pressure pain threshold.

### 3.2. Mean Differences Between the Outcomes

#### 3.2.1. Disability‐NDI

**FIGURE 2:**
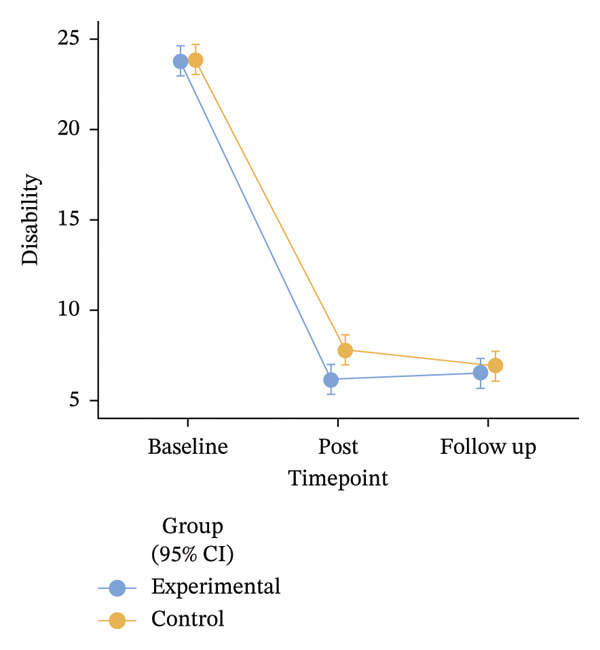
Disability scores across the timelines baseline, 6 weeks (post), and follow‐up (12 weeks).

Subjects in the experimental group exhibited an NDI score of 23.78 at baseline, 6.15 posttreatment and 6.52 at the follow‐up period, while the control group exhibited 23.89 at baseline, 7.78 posttreatment, and 6.93 at follow‐up. The results of the generalized linear model showed a significant main effect of group (*p* = 0.03, *η* *p* ^2^ = 0.01, small effect), suggesting a lower overall disability score for the experimental group than the control group (mean difference = −0.72). A large effect of time was observed (*p* < 0.001, *η* *p* ^2^ = 0.87, large effect), showing significant reductions in the disability from baseline to postintervention and follow‐up, with the scores maintained at the follow‐up also (post vs follow‐up; *p* = 0.831). There was no significant group time interaction (*p* = 0.154, *η* *p* ^2^ = 0.01, small effect) between the groups at individual time points, which were interpreted cautiously. The results of the GLM and the post hoc comparisons are presented in Tables 2 and 3 respectively. The result is graphically represented in Figure 2.

**TABLE 2:** Presents the generalized linear model results for disability using neck disability index, including the scores in the groups and across the time points.

			Mean	SE	*p* value	*η* *p* ^2^	Adj. *R* square
Group	Experimental		12.1	0.23	0.03	**0.01**	0.87
	Control		12.9	0.23			
Time	Baseline		23.83	0.29	< 0.001	**0.87**	
	Post		6.96	0.29			
	Follow up		6.72	0.29			
Group × Time	*Group*	*Time point*			0.154	**0.01**	
	Experimental	Baseline	23.78	0.41			
	Control	Baseline	23.89	0.41			
	Experimental	Post	6.15	0.41			
	Control	Post	7.78	0.41			
	Experimental	Follow up	6.52	0.41			
	Control	Follow up	6.93	0.41			

*Note:* The bold values represent the effect size.

**TABLE 3:** Post hoc analysis for disability, using neck disability index including the scores in the groups and across the time points.

Post hoc analysis for neck disability index				
Comparison‐groups				
Group	Group	Difference	df	*p* Tukey
Experimental	Control	−0.72	318	0.03

**Comparison-time point**				

**Time point**	**Time point**	**Difference**	**df**	***p* Tukey**

Baseline	Follow up	17.11	318	< 0.001
Baseline	Post	16.87	318	< 0.001
Post	Follow up	0.24	318	0.831

**Comparison-group and time points**				

Experimental	Control	−0.11	318	1.00
Baseline	Baseline			
Experimental	Control	−1.63	318	0.06
Post	Post			
Experimental	Control	−0.41	318	0.98
Follow up	Follow up			

#### 3.2.2. NPRS

**FIGURE 3:**
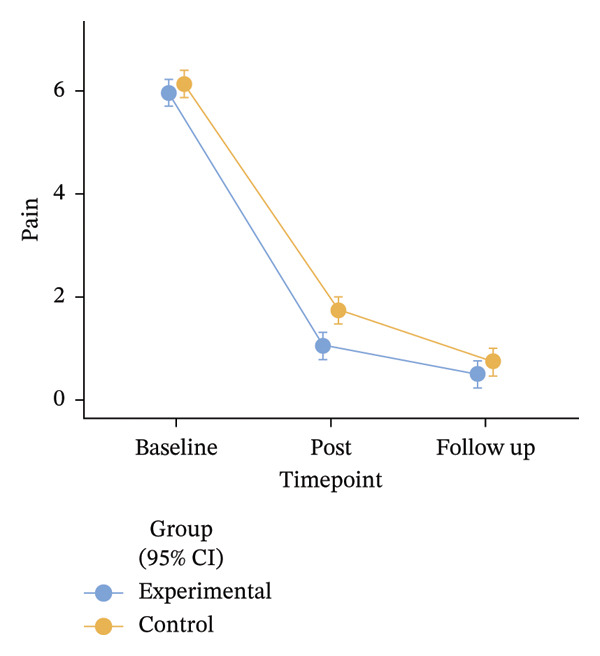
Pain scores across the timelines baseline, 6 weeks (post), and follow‐up (12 weeks).

Subjects in the experimental group exhibited a NPRS score of 5.96 at baseline, 1.05 posttreatment and 0.50 at the follow‐up period, while the control group exhibited 6.13 at baseline, 1.74 posttreatment, and 0.74 at follow‐up. Pain scores from the generalized linear model showed a significant group effect (*p* < 0.001, *η* *p* ^2^ = 0.035, small effect) with the experimental group showing lower pain scores than the control group (mean difference = −0.36). A large main effect of time was observed (*p* < 0.001, *η* *p* ^2^ = 0.861, large effect) showing reductions in the pain scores from baseline to postintervention and follow‐up timelines, indicating a continued improvement over time. There was no significant group × time interaction (*p* = 0.10, *η* *p* ^2^ = 0.014, small effect); hence, between‐group comparisons at individual time points were cautiously interpreted. The post hoc comparison suggested that the experimental group had lower scores than the control group at postintervention (mean difference = −0.69, *p* = 0.004). No significant difference was observed between the groups at the baseline (mean difference = −0.167, *p* = 1.00) and follow‐up (mean difference = −0.24, *p* = 1.00). The details of the GLM and the post hoc comparison are presented in Tables 4 and 5 respectively. The result is graphically represented in Figure 3.

**TABLE 4:** Generalized linear model results for pain using numerical pain rating scale, including the scores in the groups and across the time points.

			Mean	SE	*p* value	*η* *p* ^2^	Adj. *R* square
Group	Experimental		2.51	0.07	< 0.001	**0.035**	0.860
	Control		2.87	0.07			
Time	Baseline		6.04	0.09	< 0.001	**0.861**	
	Post		1.39	0.09			
	Follow up		0.62	0.09			
Group × Time	*Group*	*Time point*			0.10	**0.014**	
	Experimental	Baseline	5.96	0.13			
	Control	Baseline	6.13	0.13			
	Experimental	Post	1.05	0.13			
	Control	Post	1.74	0.13			
	Experimental	Follow up	0.50	0.13			
	Control	Follow up	0.74	0.13			

*Note:* The bold values represent the effect size.

**TABLE 5:** Post hoc analysis with the Tukey test for numerical pain rating scale, including the scores in the groups and across the time points.

Post hoc analysis for numerical pain rating scale				
Comparison‐groups				
Group	Group	Difference	df	*p* Tukey
Experimental	Control	−0.36	318	< 0.001

**Comparison-time point**				

**Time point**	**Time point**	**Difference**	**df**	***p* Tukey**

Baseline	Follow up	5.43	318	< 0.001
Baseline	Post	4.65	318	< 0.001
Post	Follow up	0.78	318	< 0.001

**Comparison-group and time points**				

Experimental	Control	−0.167	318	1.00
Baseline	Baseline			
Experimental	Control	−0.69	318	0.004
Post	Post			
Experimental	Control	−0.24	318	1.00
Follow up	Follow up			

### 3.3. PPT: Levator Scapulae

**FIGURE 4:**
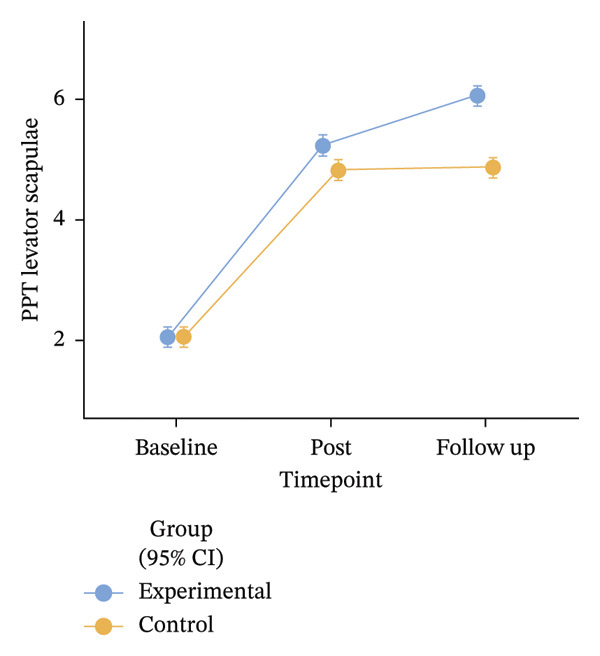
Pressure pain threshold scores of levator scapulae across the timelines baseline, 6 weeks (post), and follow‐up (12 weeks).

Subjects in the experimental group exhibited levator scapulae PPT scores of 1.08 at baseline, 5.03 posttreatment, and 6.02 at follow‐up, while the control group exhibited 1.05 at baseline, 4.49 posttreatment, and 5.88 at follow‐up. The generalized linear model for the PPT scores of the levator scapulae showed a significant group effect (*p* < 0.001, *η* *p* ^2^ = 0.006, small effect), suggesting that the experimental group had higher PPT values than the control group (mean difference = 0.53). A large effect of time was observed (*p* < 0.001, *η* *p* ^2^ = 0.95, large effect) showing improvement in the PPT scores over time from the baseline to posttreatment and follow‐up. The group × time interaction showed a significant improvement (*p* < 0.001, *η* *p* ^2^ = 0.66, large effect), suggesting that the two groups differed in their pattern over the time. The results of the post hoc analysis showed that there was no difference between the groups at the baseline (mean difference = 0.009, *p* = 1.00), but there was a significant difference in the PPT scores in the experimental group at both postintervention (mean difference = 0.40, *p* = 0.01) and the follow‐up (mean difference = 1.19, *p* < 0.001). The details of the GLM and the post hoc results are provided in Tables 6 and 7, respectively. The results are graphically represented in Figure 4.

**TABLE 6:** Generalized linear model results for the pressure pain threshold using pressure algometer of levator scapulae, including the scores in the groups and across the time points.

			Mean	SE	*p* value	*η* *p* ^2^	Adj. *R* square
Group	Experimental		4.04	0.03	< 0.001	**0.006**	0.95
	Control		3.97	0.03			
Time	Baseline		1.06	0.04	< 0.001	**0.95**	
	Post		5.01	0.04			
	Follow up		5.95	0.04			
Group × Time	*Group*	*Time point*			< 0.001	**0.66**	
	Experimental	Baseline	1.08	0.06			
	Control	Baseline	1.05	0.06			
	Experimental	Post	5.03	0.06			
	Control	Post	4.99	0.06			
	Experimental	Follow up	6.02	0.06			
	Control	Follow up	5.88	0.06			

*Note:* The bold values represent the effect size.

**TABLE 7:** Post hoc analysis with the Tukey test for the pressure pain threshold using the pressure algometer of the levator scapulae, including the scores in the groups and across the time points.

Post hoc analysis for pain pressure threshold‐levator scapulae				
Comparison‐groups				
Group	Group	Difference	df	*p* Tukey
Experimental	Control	0.53	318	< 0.001

**Comparison-time point**				

**Time point**	**Time point**	**Difference**	**df**	***p* Tukey**

Baseline	Follow up	−3.40	318	< 0.001
Baseline	Post	−2.97	318	< 0.001
Post	Follow up	−0.44	318	< 0.001

**Comparison-group and time points**				

Experimental	Control	0.009	318	1.00
Baseline	Baseline			
Experimental	Control	0.40	318	0.01
Post	Post			
Experimental	Control	1.19	318	< 0.001
Follow up	Follow up			

### 3.4. PPT Upper Trapezius

**FIGURE 5:**
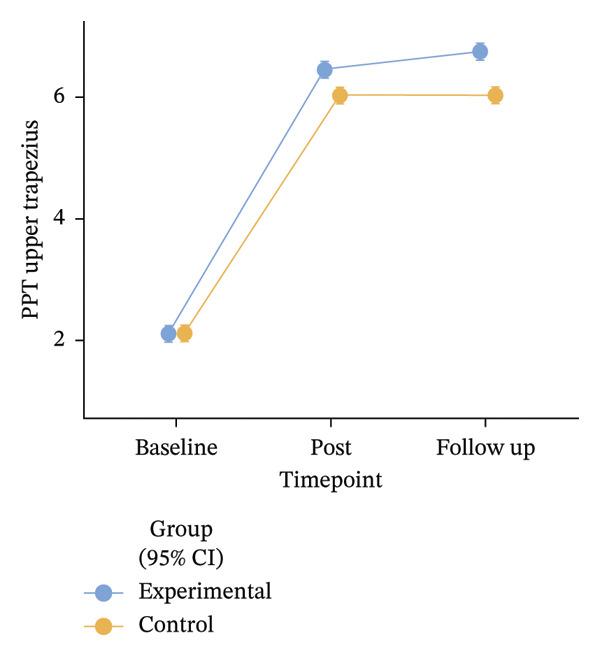
pressure pain threshold scores using pressure algometer of the upper trapezius across the timelines baseline, 6 weeks (post), and follow‐up (12 weeks).

Subjects in the experimental group exhibited upper trapezius PPT scores of 2.10 at baseline, 6.45 posttreatment and 6.75 at the follow‐up period, while the control group exhibited 2.10 at baseline, 6.03 posttreatment, and 6.02 at follow‐up. There was a significant effect between the groups (*p* < 0.001, *η* *p* ^2^ = 0.16, large effect) in the upper trapezius PPT scores. The results suggest that the PPT scores in the experimental group were higher than those of the control group (mean difference = 0.38). A large effect of time was observed (*p* < 0.001, *η* *p* ^2^ = 0.95, large effect), suggesting a progressive improvement in the PPT scores from baseline to postintervention and follow‐up. A significant difference was also observed in the group × time interaction (*p* < 0.001, *η* *p* ^2^ = 0.10, large effect), indicating that the groups differed in their pattern over the time. There was no significant difference between the groups at the baseline (mean difference = −0.007, *p* = 1.00), but the experimental group showed higher PPT scores at postintervention (mean difference = 0.42, *p* < 0.001) and at the follow‐up (mean difference = 0.73, *p* < 0.001). The details of the GLM analysis and the post hoc analysis are presented in Tables 8 and 9, respectively. The results are graphically represented in Figure 5.

**TABLE 8:** Generalized linear model results for the pressure pain threshold using the pressure algometer of upper trapezius, including the scores in the groups and across the time points.

			Mean	SE	*p* value	*η* *p* ^2^	Adj. *R* square
Group	Experimental		5.10	0.03	< 0.001	**0.16**	0.95
	Control		4.72	0.03			
Time	Baseline		2.10	0.04	< 0.001	**0.95**	
	Post		6.24	0.04			
	Follow up		6.39	0.04			
Group × Time	*Group*	*Time point*			< 0.001	**0.10**	
	Experimental	Baseline	2.10	0.05			
	Control	Baseline	2.10	0.05			
	Experimental	Post	6.45	0.05			
	Control	Post	6.03	0.05			
	Experimental	Follow up	6.75	0.05			
	Control	Follow up	6.02	0.05			

*Note:* The bold values represent the effect size.

**TABLE 9:** Post hoc analysis with the Tukey test for the pressure pain threshold of upper trapezius using pressure algometer, including the scores in the groups and across the time points.

Post hoc analysis for pain pressure threshold‐upper trapezius				
Comparison‐groups				
Group	Group	Difference	df	*p* Tukey
Experimental	Control	0.38	318	< 0.001

**Comparison-time point**				

**Time point**	**Time point**	**Difference**	**df**	***p* Tukey**

Baseline	Follow up	−4.29	318	< 0.001
Baseline	Post	−4.14	318	< 0.001
Post	Follow up	−0.15	318	0.04

**Comparison-group and time points**				

Experimental	Control	−0.007	318	1.00
Baseline	Baseline			
Experimental	Control	0.42	318	< 0.001
Post	Post			
Experimental	Control	0.73	318	< 0.001
Follow up	Follow up			

### 3.5. PPT Spine

**FIGURE 6:**
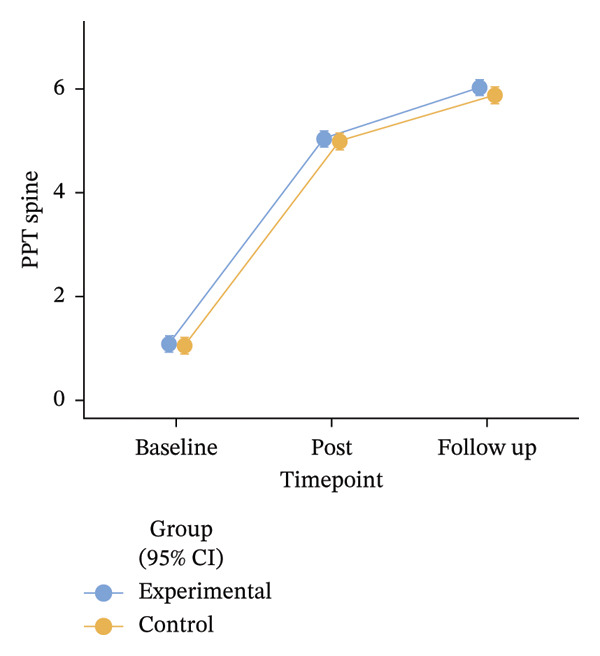
Pressure pain threshold scores of the spine across the timelines baseline, 6 weeks (post), and follow‐up (12 weeks).

Subjects in the experimental group exhibited cervical spine PPT scores of 2.10 at baseline, 6.45 posttreatment and 6.75 at the follow‐up period, while the control group exhibited 2.10 at baseline, 6.03 posttreatment, and 6.02 at follow‐up. The generalized linear model for the PPT spine scores showed that there was no significant group effect (*p* = 0.17, *η* *p* ^2^ = 0.16), large effect and no significant group × time interaction (*p* = 0.66, *η* *p* ^2^ = 0.10, large effect), suggesting that the groups did not differ in their pattern of change over time. Despite the large effect size, these effects did not reach statistical significance, which can be attributed to the dominant effect of time. A large effect of time was observed (*p* < 0.001, *η* *p* ^2^ = 0.95, large effect), suggesting that the PPT scores improved over time from baseline to posttreatment and the follow‐up. The results of the post hoc analysis showed that there was no significant difference between the groups in the baseline (mean difference = 0.03, *p* = 0.99), postintervention (mean difference = 0.05, *p* = 0.99) and follow‐up (mean difference = 0.14, *p* = 0.65). The details of the GLM analysis and the post hoc analysis are presented in Tables 10 and 11, respectively. The result is graphically represented in Figure 6.

**TABLE 10:** Generalized linear model results for the pressure pain threshold using the pressure algometer threshold of the spine, including the scores in the groups and across the time points.

			Mean	SE	*p* value	*η* *p* ^2^	Adj. *R* square
Group	Experimental		5.10	0.03	0.17	**0.16**	0.95
	Control		4.72	0.03			
Time	Baseline		2.10	0.04	< 0.001	**0.95**	
	Post		6.24	0.04			
	Follow up		6.39	0.04			
Group × Time	*Group*	*Time point*			0.66	**0.10**	
	Experimental	Baseline	2.10	0.05			
	Control	Baseline	2.10	0.05			
	Experimental	Post	6.45	0.05			
	Control	Post	6.03	0.05			
	Experimental	Follow up	6.75	0.05			
	Control	Follow up	6.02	0.05			

*Note:* The bold values represent the effect size.

**TABLE 11:** Post hoc analysis with the Tukey test of the pressure pain threshold using the pressure algometer of the spine, including the scores across the groups and time points.

Post hoc analysis for pain pressure threshold‐spine				
Comparison‐groups				
Group	Group	Difference	df	*p* Tukey
Experimental	Control	0.07	318	0.18

**Comparison-time point**				

**Time point**	**Time point**	**Difference**	**df**	***p* Tukey**

Baseline	Follow up	−4.89	318	< 0.001
Baseline	Post	−3.95	318	< 0.001
Post	Follow up	−0.94	318	< 0.001

**Comparison-group and time points**				

Experimental	Control	0.03	318	0.99
Baseline	Baseline			
Experimental	Control	0.05	318	0.99
Post	Post			
Experimental	Control	0.14	318	0.65
Follow up	Follow up			

A secondary analysis was performed to determine the effects of sex and age on pain and disability. Linear regression was performed to determine the effects of sex and age on pain. The analysis revealed no significant effect of age (*p* = 0.49) or sex (*p* = 0.61) on pain, and no significant effect of age (*p* = 0.46) or sex (*p* = 0.34) on disability.

The subjects were assessed for compliance with the exercises via the Correctness of Exercise Performance Scale. However, 2% of the subjects reported a decline in exercise performance during the follow‐up period. The subjects reported an average adherence rate of 82%. There were no adverse events noted at any point during the trial.

## 4. Discussion

This study examined the effects of scapular‐focused interventions on pain and disability in patients with neck pain with mobility deficits.

### 4.1. Disability

The results of the study showed that the disability scores improved among both the groups across all the timelines, with a similar pattern of improvement over time. The between‐group differences in the NDI score in both groups (scapula‐focused group‐17.63, neck intervention group‐16.11) at 6 weeks reached a minimum detectable change of 10 and a minimum clinical importance difference of 5. These results are similar to the findings of recent studies in which the effects of scapular interventions on neck pain were examined [39–42]. Yildiz et al. [19] also reported a greater effect (*r* = 0.69) for the NDI.

Scapula‐focused interventions are directed at strengthening the scapular and periscapular muscles. The interventions were also aimed at enhancing mobility and were structured to minimize the load on the upper trapezius and levator scapula while the exercises were performed. This might have led to improved positioning of the scapula and the establishment of the optimal length tension relationship [43]. This could potentially have improved the activities of daily living and hence reduced disability. However, the control group also exhibited similar findings.

The possible mechanism for the reduction in pain may involve improvements in the strength of the neck muscles and neck posture. The neuromuscular improvement with the stabilization exercises could potentially reduce the load on the cervical spine [44]. This can potentially affect the balance of the cervical structures, which may lead to mechanical changes in the neck and associated structures. However, the neck acts as a link between the head and torso and receives muscular attachments from the spine and appendicular skeleton. The scapula acts as a link between these structures and aids in load transmission [45]. Any alterations in position may alter the length‐tension relationship of the axioscapular and the cervicoscapular muscles, causing an abnormal distribution of load in the cervical spine and thereby causing neck pain [6]. Hence, scapular exercises are beneficial, as they help in mobilizing and stabilizing the scapula, establishing a strong kinetic link between the neck and the torso. Hence, focused scapular interventions alone may be as effective for reducing disability as neck strengthening exercises.

Musculoskeletal pain is frequently associated with disability [46]. The subjects in the current study were identified as having a moderate level of disability. The acuity of the symptoms could be the reason for the moderate disability presented. Disability and pain duration are directly associated, and hence, the trend in the decline of the disability scores with the pain scores is sensible. Both interventions were targeted at reducing pain by balancing the force on the cervical and scapular regions and thereby reducing pain, which might have led to a reduction in disability scores. Along with the theories that muscular enhancement improves the scores of disability, another proposed mechanism for the reduction in disability could be the perceived stress reduction at the amygdala and the cingulate complex and exercise‐induced hypoalgesia [47–49]. Overall, when pain is reduced, disability might also change in a positive manner, leading to improvements in NDI scores [50].

### 4.2. Pain

The results of the present study revealed that there was no significant difference between the groups for the NPRS pain scores. Both groups demonstrated clinically meaningful improvements exceeding the MCID and MDC. The experimental group exhibited an improvement of 4.91 at 6 weeks and 5.46 at 12 weeks. The control group revealed improvements of 4.39 at the 6^th^ week and 5.39 at the 12^th^ week. This suggests a similar pattern of improvement across the groups over time. These values are greater than the MCID and MDC values of NPRS, which are 1.3–2 and 1.4–2, respectively. A moderate effect size was observed between the groups.

The findings of this study are similar to findings of recent studies [19, 40, 41, 43] on the effects of scapular exercises on neck pain. These studies also reported a decrease in pain intensity at 4–6 weeks. Yildiz et al. [19] reported a significant effect of time, but there was no significant difference between the groups. However, these studies did not perform any follow‐up after treatment, which makes comparisons with the findings of the present study difficult. The results of the current study suggest that scapular interventions are as effective as neck exercise over time. Both groups showed a comparable reduction in the pain score over time, consistent with nonsignificant group × time interaction. This suggests that scapular interventions and the neck exercises were equally effective in reducing pain.

The possible mechanism which led to reduced pain in the subjects who performed scapula‐focused interventions could be due to (1) the reduced load on the cervicoscapular muscles, which may reduce the passive stretch on these muscle groups during the active rotation of the neck; (2) reduced load on the cervical structures; and (3) reduced stress on the nervous structures around the neck and scapula [51]. Active neck restricted movement is mostly associated with the neck pain with mobility deficits subgroup. Hence, regaining normal neck movements with synchronized activity of the scapular muscles due to their attachment to the cervical and scapular spine might have led to reduced pain.

Neck exercises, which are also focused on stabilizing the cervical muscles, could have led to a reduced load on the cervical spine structures and thereby reduced pain impulses. Possible mechanisms include increased activity of the motor pathways, which in turn inhibits the pain centers in the central nervous system, and activation of the deep cervical muscles, which may result in reduced tension on the superficial muscles, stimulating mechanoreceptors and increasing sensory activity, thereby potentially inhibiting pain pathways.

### 4.3. PPT Scores

The PPT for upper trapezius and levator scapulae improved in both groups across all timelines. A significant group × time interaction was observed for both muscles, with the scapular intervention group demonstrating greater improvements compared to the neck exercise group, particularly postintervention and follow‐up. The PPT over the cervical spine improved over time; however, there was no significant group × time interaction. Statistically, a significant group × time interaction was observed in PPT for both muscles, showing improvement between the groups over time. These results suggested that both the groups improved similarly. However, for the spine, only the main effect of time was observed. A clinical improvement was observed in the PPT values for upper trapezius, levator scapulae, and spine: 32.5%, 21%, and 32.5%, respectively that falls within the MCID and MDC of 33% and 10%–15%, respectively. This confirms that both the groups achieved clinical improvements by the end of the 6^th^ week.

The findings of this study are contradictory to the findings of Srikaranaj and Kanlayanaphotoron [52] and Bobos et al. [53]. The difference in the observed results of the findings of Srikaranaj and Kanlayanaphotoron [52] could be due to the difference in the interventions used targeting the scapula. The study was conducted with active correction of the scapula, holding the scapula in the resting position. Additionally, Bobos et al. [53] reported that there was no significant improvement in the PPT at myofascial trigger points. The possible reason for the observed results could be the differences in the exercise interventions.

There was a significant reduction in the PPT values in the upper trapezius, levator scapulae, and spinous process of the cervical spine among the groups and between the timelines. No studies to date have specifically assessed the effects of scapular interventions on the PPT of the scapular muscles. Studies have shown that the activation of the upper trapezius and levator scapulae muscles is greater during overhead activities or during specific job tasks [54, 55]. This increases the tension on the upper trapezius and levator scapula, which in turn may impact the spine, causing increased sensitivity in these regions. The exercises included in the current study were designed such that the activation of the upper trapezius and levator scapulae was minimal. The exercises in the current study also involved antagonists of scapular elevation. This focus on the scapula depressors and the retractors would have made the scapula into the ideal resting position and thereby may have established the ideal length‐tension relationship in the spinoscapular muscles. This might be the reason for the increased PPT values over the upper trapezius and levator scapulae muscles. The reduced load on these muscles may indirectly reduce the load on the cervical spine. This could potentially lead to improved PPTs over the cervical spine. However, as the current study did not investigate kinematic or EMG parameters, the proposed mechanism remains hypothetical and warrants further investigation.

The neck exercise group also exhibited a significant reduction in PPT values. This might be due to the activation of deep cervical neck muscles, which affect the posture of the neck and thereby achieve a balanced forced distribution on the cervical spine. However, the results suggest that the threshold values were greater for the scapular group for the levator scapulae and upper trapezius muscles. This could be due to the antagonistic action of the muscles targeted in the intervention, especially the lower trapezius, serratus anterior, and middle trapezius.

The current study had a 12 week follow‐up, which provides valuable insight into the long‐term effects of scapular interventions. This was evident across all the outcomes, suggesting that the benefits of scapular interventions may be as beneficial as the neck‐directed exercises. This could be due to the progressive development of scapular motor control through the scapular interventions. From a clinical perspective, it could be advocated that scapular‐focused interventions may offer long‐term benefits for individuals with neck pain with mobility deficits.

### 4.4. Strengths of the Study

This study is the first to examine the isolated effects of scapular exercises on pain and disability in subjects with neck pain with mobility deficits. This intervention adds to clinical practice with easy administration and cost effectiveness. Additionally, the current study assessed the influence of the scapula on the range of motion of the neck by performing scapular assistance and scapular retraction tests.

### 4.5. Limitations

The limitations of the study include that the subjects were assessed for scapular involvement at baseline, but the degree of correction or the position of the scapula after the interventions was not assessed at any point in the study. Additionally, a baseline difference in the lower trapezius strength was observed between the groups, despite randomization. However, this variable was not included in the primary analysis, and hence it might have affected the between‐group comparisons at follow‐up. The study did not include a no‐treatment control group, which might mean the subjects could have improved simply through the passage of time. The study did not assess biochemical markers for the changes observed in the muscles. Although adherence to exercise was measured, the subjective nature of the outcome has its own bias.

### 4.6. Future Recommendations

The role of the scapula in other clinical conditions, such as neck pain with radiculopathy, neck pain with motor control deficits, and neck pain with headache, has not been studied. Future studies can explore the role of screening scapular involvement and scapular strengthening exercises in such conditions. Studies should also explore the optimal dose of scapular exercises in neck pain with mobility deficits and other neck conditions.

## 5. Conclusion

The results of the study suggest that both scapular‐focused interventions and neck exercises led to clinically meaningful improvements in pain and disability and PPT over time. Pain and disability improved similarly in both groups, suggesting that the scapular interventions are as effective as the neck exercises for these outcomes. However, there was not a no‐treatment control group, so these results should be interpreted with caution. The scapular intervention group demonstrated greater improvements in PPT values of the upper trapezius and levator scapulae, particularly at postintervention and follow‐up, indicating a superior effect of scapular‐focused interventions in reducing cervicoscapular muscle sensitivity. As scapula repositioning helps in improving the symptoms, scapula‐focused interventions can be considered predominantly for neck pain with mobility deficits, either in isolation or in combination with neck exercises.

## Author Contributions

Nithin Prakash: data curation, methodology, formal analysis, investigation, methodology, and writing–original draft.

Karvannan Harikesavan: conceptualization, methodology, project administration, supervision, validation, visualization, and writing–review and editing.

Joshua Cleland: conceptualization, validation, methodology, supervision, and writing–review and editing.

Hemant K. Kalyan: resources, methodology, visualization, supervision, and writing–review and editing.

## Funding

No funding was received for this manuscript.

## Disclosure

The study protocol for this trial was previously published in Reviews on Recent Clinical Trials in November 2023 [4].

## Ethics Statement

The ethical committee approval for the study was obtained from the Ethics Committee, Manipal Hospitals, Bangalore. Informed consent was obtained from the participants before enrolling them for the trial. The study was conducted in accordance with the Declaration of Helsinki.

## Conflicts of Interest

The authors declare no conflicts of interest.

## Supporting Information

Additional supporting information can be found online in the Supporting Information section.

## Supporting information


**Supporting Information** The details of the exercises for both the experimental and control group are provided in the supporting information.

## Data Availability

Data are available on request from the corresponding author.

## References

[1] Merskey H. , Bogduk N. , Antony A. , and Singh R. , Slipman C. W. , Derby R. , Simeone F. A. et al., Medical Causes of Neck Pain, Classification of Chronic Pain: Descriptions of Chronic Cpain Syndromes and Definition of Pain Terms, 1994, 2nd edition, IASP Press, Seattle, 103–111, 10.1016/B978-0-7216-2872-1.50052-2.

[2] Kazeminasab S. , Nejadghaderi S. A. , Amiri P. et al., Neck Pain: Global Epidemiology, Trends and Risk Factors, BMC Musculoskeletal Disorders. (2022) 23, no. 1, 10.1186/s12891-021-04957-4. PMC8725362 34980079

[3] Blanpied P. R. , Gross A. R. , Elliott J. M. et al., Neck Pain: Revision 2017: Clinical Practice Guidelines Linked to the International Classification of Functioning, Disability and Health From the Orthopaedic Section of the American Physical Therapy Association, Journal of Orthopaedic & Sports Physical Therapy. (2017) 47, no. 7, A1–A83, 10.2519/jospt.2017.0302. 28666405

[4] Prakash N. , Cleland J. , and Harikesavan K. , Effect of Scapula Focused Interventions on Pain and Disability in Neck Pain With Mobility Deficits-Protocol for a Single Blinded Randomized Controlled Trial, Reviews on Recent Clinical Trials. (2023) 18, no. 4, 282–287, 10.2174/1574887118666230519155631. 38192198

[5] Hogg-Johnson S. , van der Velde G. , Carroll L. J. et al., The Burden and Determinants of Neck Pain in the General Population: Results of the Bone and Joint Decade 2000–2010 Task Force on Neck Pain and Its Associated Disorders, Journal of Manipulative and Physiological Therapeutics. (2009) 32, no. 2 Suppl, S46–S60, 10.1016/j.jmpt.2008.11.010. 19251074

[6] Cagnie B. , Struyf F. , Cools A. , Castelein B. , Danneels L. , and O’leary S. , The Relevance of Scapular Dysfunction in Neck Pain: A Brief Commentary, Journal of Orthopaedic & Sports Physical Therapy. (2014) 44, no. 6, 435–439, 10.2519/jospt.2014.5038. 24816504

[7] Sciascia A. and Ben Kibler W. , Current Concepts: Scapular Dyskinesis, British Journal of Sports Medicine. (2010) 44, no. 5, 300–305, 10.1136/bjsm.2009.058834. 19996329

[8] Ibrahim T. , Cools A. , and Duzgun I. , Clinical Biomechanics Alterations in the 3-Dimensional Scapular Orientation in Patients With Non-Speci Fi C Neck Pain, Clinical Biomechanics. (2019) 70, 97–106, 10.1016/j.clinbiomech.2019.08.007. 31450180

[9] Kim S. R. , Kang M. H. , Bahng S. Y. et al., Correlation Among Scapular Asymmetry, Neck Pain, and Neck Disability Index (NDI) in Young Women With Slight Neck Pain, Journal of Physical Therapy Science. (2016) 28, no. 5, 1508–1510, 10.1589/jpts.28.1508. 27313361 PMC4905900

[10] Falla D. , Bilenkij G. , and Jull G. , Patients With Chronic Neck Pain Demonstrate Altered Patterns of Muscle Activation During Performance of a Functional Upper Limb Task, Spine. (2004) 29, no. 13, 1436–1440, 10.1097/01.brs.0000128759.02487.bf. 15223935

[11] Johnston V. , Jull G. , Darnell R. , Jimmieson N. L. , and Souvlis T. , Alterations in Cervical Muscle Activity in Functional and Stressful Tasks in Female Office Workers With Neck Pain, European Journal of Applied Physiology. (2008) 103, no. 3, 253–264, 10.1007/s00421-008-0696-8. 18293008

[12] Helgadottir H. , Kristjansson E. , Einarsson E. , Karduna A. , and Jonsson H. , Altered Activity of the Serratus Anterior During Unilateral Arm Elevation in Patients With Cervical Disorders, Journal of Electromyography and Kinesiology. (2011) 21, no. 6, 947–953, 10.1016/j.jelekin.2011.07.007. 21889362

[13] Hons E. Z. B. , Mphty G. J. , Bphty V. J. , and Mphty S. O. L. , Altered Trapezius Muscle Behavior in Individuals With Neck Pain and Clinical Signs of Scapular Dysfunction, Journal of Manipulative and Physiological Therapeutics, 35, 346–353, 10.1016/j.jmpt.2012.04.011. 22608287

[14] Johnston V. , Jull G. , Souvlis T. , and Jimmieson N. L. , Neck Movement and Muscle Activity Characteristics in Female Office Workers With Neck Pain, Spine. (2008) 33, no. 5, 555–563, 10.1097/brs.0b013e3181657d0d. 18317202

[15] Lluch E. , Arguisuelas M. D. , Calvente Quesada O. et al., Immediate Effects of Active Versus Passive Scapular Correction on Pain and Pressure Pain Threshold in Patients With Chronic Neck Pain, Journal of Manipulative and Physiological Therapeutics. (2014) 37, no. 9, 660–666, 10.1016/j.jmpt.2014.08.007. 25282679

[16] Ha S. , Kwon O. , Yi C. , Jeon H. , and Lee W. , Effects of Passive Correction of Scapular Position on Pain, Proprioception, and Range of Motion in Neck-Pain Patients With Bilateral Scapular Downward-Rotation Syndrome, Manual Therapy. (2011) 16, no. 6, 585–589, 10.1016/j.math.2011.05.011. 21705260

[17] Park S.-H. and Lee M.-M. , Effects of Lower Trapezius Strengthening Exercises on Pain, Dysfunction, Posture Alignment, Muscle Thickness and Contraction Rate in Patients With Neck Pain; Randomized Controlled Trial, Medical Science Monitor: International Medical Journal of Experimental and Clinical Research. (2020) 26, 10.12659/MSM.920208. PMC7115121 32202262

[18] Cavalcanti I. F. , Antonino G. B. , do Monte-Silva K. K. , Guerino M. R. , Ferreira A. P. D. L. , and das Graças Rodrigues de Araújo M. , Global Postural Re-Education in Non-Specific Neck and Low Back Pain Treatment: A Pilot Study, Journal of Back and Musculoskeletal Rehabilitation. (2020) 33, no. 5, 823–828, 10.3233/BMR-181371. 31929138

[19] Yildiz T. I. , Turgut E. , and Duzgun I. , Neck and Scapula-Focused Exercise Training on Patients With Nonspecific Neck Pain: A Randomized Controlled Trial, Journal of Sport Rehabilitation. (2018) 27, no. 5, 403–412, 10.1123/jsr.2017-0024. 28605288

[20] Ashwini T. M. , Karvannan H. , and Prem V. , Effects of Movement Impairment Based Treatment in the Management of Mechanical Neck Pain, Journal of Bodywork and Movement Therapies. (2018) 22, no. 2, 534–539, 10.1016/j.jbmt.2017.07.007. 29861262

[21] Seo Y. G. , Park W. H. , Lee C. S. et al., Is Scapular Stabilization Exercise Effective for Managing Nonspecific Chronic Neck Pain?: A Systematic Review, Asian Spine Journal. (2020) 14, no. 1, 122–129, 10.31616/asj.2019.0055. 31668049 PMC7010515

[22] Consort Group , CONSORT 2010 Checklist, 2010, CONSORT, https://www.consort-statement.org.

[23] Reinold M. M. , Escamilla R. , and Wilk K. E. , Current Concepts in the Scientific and Clinical Rationale Behind Exercises for Glenohumeral and Scapulothoracic Musculature, Journal of Orthopaedic & Sports Physical Therapy. (2009) 39, no. 2, 105–117, 10.2519/jospt.2009.2835. 19194023

[24] Schory A. , Bidinger E. , Wolf J. , and Murray L. , A Systematic Review of the Exercises That Produce Optimal Muscle Ratios of the Scapular Stabilizers in Normal Shoulders, International Journal of Sports Physical Therapy. (2016) 11, no. 3, 321–336. 27274418 PMC4886800

[25] Donatelli R. A. and Wooden M. J. , Orthopaedic Physical Therapy, 2009, Elsevier Health Sciences.

[26] Sarig-Bahat H. , Evidence for Exercise Therapy in Mechanical Neck Disorders, Manual Therapy. (2003) 8, no. 1, 10–20, 10.1054/math.2002.0480. 12586557

[27] Kaka B. , Ogwumike O. O. , Adeniyi A. F. , Maharaj S. S. , Ogunlade S. O. , and Bello B. , Effectiveness of Neck Stabilisation and Dynamic Exercises on Pain Intensity, Depression and Anxiety Among Patients With Non-Specific Neck Pain: A Randomised Controlled Trial, Scandinavian Journal of Pain. (2018) 18, no. 2, 321–331, 10.1515/sjpain-2017-0146. 29794305

[28] Friedrich M. , Cermak T. , and Maderbacher P. , The Effect of Brochure Use Versus Therapist Teaching on Patients Performing Therapeutic Exercise and on Changes in Impairment Status, Physical Therapy. (1996) 76, no. 10, 1082–1088, 10.1093/ptj/76.10.1082. 8863761

[29] Newman-Beinart N. A. , Norton S. , Dowling D. et al., The Development and Initial Psychometric Evaluation of a Measure Assessing Adherence to Prescribed Exercise: The Exercise Adherence Rating Scale (EARS), Physiotherapy. (2017) 103, no. 2, 180–185, 10.1016/j.physio.2016.11.001. 27913064

[30] Young I. A. , Dunning J. , Butts R. , Mourad F. , and Cleland J. A. , Reliability, Construct Validity, and Responsiveness of the Neck Disability Index and Numeric Pain Rating Scale in Patients With Mechanical Neck Pain Without Upper Extremity Symptoms, Physiotherapy Theory and Practice. (2019) 35, no. 12, 1328–1335, 10.1080/09593985.2018.1471763. 29856244

[31] MacDermid J. C. , Walton D. M. , Avery S. et al., Measurement Properties of the Neck Disability Index: A Systematic Review, Journal of Orthopaedic & Sports Physical Therapy. (2009) 39, no. 5, 400–C12, 10.2519/jospt.2009.2930. 19521015

[32] Cleland J. A. , Childs J. D. , and Whitman J. M. , Psychometric Properties of the Neck Disability Index and Numeric Pain Rating Scale in Patients With Mechanical Neck Pain, Archives of Physical Medicine and Rehabilitation. (2008) 89, no. 1, 69–74, 10.1016/j.apmr.2007.08.126. 18164333

[33] Bhattacharyya A. , Hopkinson L. D. , Nolet P. S. , and Srbely J. , The Reliability of Pressure Pain Threshold in Individuals With Low Back or Neck Pain: A Systematic Review, British Journal of Pain. (2023) 17, no. 6, 579–591, 10.1177/20494637231196647. 37969131 PMC10642499

[34] Diep D. , Chen K. J. Q. , and Kumbhare D. , Ultrasound-Guided Interventional Procedures for Myofascial Trigger Points: A Systematic Review, Regional Anesthesia and Pain Medicine. (2021) 46, no. 1, 73–80, 10.1136/rapm-2020-101898. 33159004

[35] Walton D. M. , Macdermid J. C. , Nielson W. , Teasell R. W. , Nailer T. , and Maheu P. , A Descriptive Study of Pressure Pain Threshold at 2 Standardized Sites in People With Acute or Subacute Neck Pain, Journal of Orthopaedic & Sports Physical Therapy. (2011) 41, no. 9, 651–657, 10.2519/jospt.2011.3667. 21885907

[36] Helgadottir H. , Kristjansson E. , Mottram S. , Karduna A. , and Jonsson H.Jr., Altered Scapular Orientation During Arm Elevation in Patients With Insidious Onset Neck Pain and Whiplash-Associated Disorder, Journal of Orthopaedic & Sports Physical Therapy. (2010) 40, no. 12, 784–791, 10.2519/jospt.2010.3405. 20972341

[37] Keating L. , Lubke C. , Powell V. , Young T. , Souvlis T. , and Jull G. , Mid-Thoracic Tenderness: A Comparison of Pressure Pain Threshold Between Spinal Regions, Asymptomatic Subjects, Manual Therapy. (2001) 6, no. 1, 34–39, 10.1054/math.2000.0377. 11243907

[38] Jamovi-Open Statistical Software for the Desktop and Cloud, 2025, https://www.jamovi.org/.

[39] Lee K.-S. , Effect of a Five-Week Scapular Correction Exercise in Patients with Chronic Mechanical Neck Pain, Journal of Korean Physical Therapy. (2020) 32, no. 2, 126–131, 10.18857/jkpt.2020.32.2.126.

[40] Kang T. and Kim B. , Cervical and Scapula-Focused Resistance Exercise Program Versus Trapezius Massage in Patients With Chronic Neck Pain: A Randomized Controlled Trial, Medicine. (2022) 101, no. 39, 10.1097/MD.0000000000030887. PMC9524908 36181044

[41] Im B. , Kim Y. , Chung Y. , and Hwang S. , Effects of Scapular Stabilization Exercise on Neck Posture and Muscle Activation in Individuals With Neck Pain and Forward Head Posture, Journal of Physical Therapy Science. (2015) 28, no. 3, 951–955, 10.1589/jpts.28.951. PMC4842472 27134391

[42] Lee H. , Hübscher M. , Moseley G. L. et al., How Does Pain Lead to Disability? A Systematic Review and Meta-Analysis of Mediation Studies in People With Back and Neck Pain, Pain. (2015) 156, no. 6, 988–997, 10.1097/j.pain.0000000000000146. 25760473

[43] Javdaneh N. , Ambroży T. , Barati A. H. , Mozafaripour E. , and Rydzik Ł. , Focus on the Scapular Region in the Rehabilitation of Chronic Neck Pain Is Effective in Improving the Symptoms: A Randomized Controlled, Trial, JCM. (2021) 10, no. 16, 10.3390/jcm10163495. PMC8397110 34441791

[44] Celenay S. T. , Akbayrak T. , and Kaya D. O. , A Comparison of the Effects of Stabilization Exercises Plus Manual Therapy to Those of Stabilization Exercises Alone in Patients With Nonspecific Mechanical Neck Pain: A Randomized Clinical Trial, Journal of Orthopaedic & Sports Physical Therapy. (2016) 46, no. 2, 44–55, 10.2519/jospt.2016.5979. 26755405

[45] Sanchez H. M. and De Morais Sanchez E. G. , Scapular Dyskinesis: Biomechanics, Evaluation and Treatment, IPMRJ. (2018) 3, no. 6, 10.15406/ipmrj.2018.03.00157.038.

[46] Vargas-Prada S. and Coggon D. , Psychological and Psychosocial Determinants of Musculoskeletal Pain and Associated Disability, Best Practice & Research Clinical Rheumatology. (2015) 29, no. 3, 374–390, 10.1016/j.berh.2015.03.003. 26612236 PMC4668591

[47] Smith B. E. , Hendrick P. , Bateman M. et al., Musculoskeletal Pain and Exercise—Challenging Existing Paradigms and Introducing New, British Journal of Sports Medicine. (2019) 53, no. 14, 907–912, 10.1136/bjsports-2017-098983. 29925503 PMC6613745

[48] Naugle K. M. , Fillingim R. B. , and Riley J. L. , A Meta-Analytic Review of the Hypoalgesic Effects of Exercise, Journal of Pain. (2012) 13, no. 12, 1139–1150, 10.1016/j.jpain.2012.09.006. 23141188 PMC3578581

[49] Cox L. G. W. , Savur K. T. , De Nardis R. J. , and Iles R. A. , Progressive Resistance Exercise for Improving Pain and Disability in Chronic Neck Pain: A Case Series, Physiotherapy Research International. (2020) 25, no. 4, 10.1002/pri.1863. 32648340

[50] Luque-Suarez A. , Martinez-Calderon J. , and Falla D. , Role of Kinesiophobia on Pain, Disability and Quality of Life in People Suffering From Chronic Musculoskeletal Pain: A Systematic Review, British Journal of Sports Medicine. (2019) 53, no. 9, 554–559, 10.1136/bjsports-2017-098673. 29666064

[51] Van Dillen L. R. , McDonnell M. K. , Susco T. M. , and Sahrmann S. A. , The Immediate Effect of Passive Scapular Elevation on Symptoms With Active Neck Rotation in Patients With Neck Pain, Clinical Journal of Pain. (2007) 23, no. 8, 641–647, 10.1097/AJP.0b013e318125c5b6. 17885341

[52] Srikrajang S. and Kanlayanaphotporn R. , Effects of Active Scapular Correction on Cervical Range of Motion, Pain, and Pressure Pain Threshold in Patients With Chronic Neck Pain and Depressed Scapula: A Randomized Controlled Trial, Journal of Manual & Manipulative Therapy. (2023) 31, no. 1, 24–31, 10.1080/10669817.2022.2077515. 35588354 PMC9848379

[53] Bobos P. , Billis E. , Papanikolaou D.-T. , Koutsojannis C. , and MacDermid J. C. , Does Deep Cervical Flexor Muscle Training Affect Pain Pressure Thresholds of Myofascial Trigger Points in Patients With Chronic Neck Pain? A Prospective Randomized Controlled Trial, Rehabilitation Research and Practice 2016. (2016) 2016, 1–8, 10.1155/2016/6480826. PMC5136630 27990302

[54] Falla D. L. , Jull G. A. , and Hodges P. W. , Patients With Neck Pain Demonstrate Reduced Electromyographic Activity of the Deep Cervical Flexor Muscles During Performance of the Craniocervical Flexion Test, Spine. (2004) 29, no. 19, 2108–2114, 10.1097/01.brs.0000141170.89317.0e. 15454700

[55] Moon S. E. and Kim Y. K. , Neck and Shoulder Pain With Scapular Dyskinesis in Computer Office Workers, Medicina. (2023) 59, no. 12, 10.3390/medicina59122159. PMC10744820 38138262

