# Supplementary material for: Effect of Scapular‐Focused Interventions on Pain and Disability in Neck Pain With Mobility Deficits: A Randomized Controlled Trial
Source: Pain Res Manag. 2026 Jul 16;2026:3143187. doi: 10.1155/prm/3143187 (PMC13374109; doi:10.1155/prm/3143187)
Supplement: Supplementary file 1 — Supporting Information The details of the exercises for both the experimental and control group are provided in the supporting information. [file PRM-2026-3143187-s001.docx]

**SUPPLEMENTARY MATERIAL**

**SCAPULAR FOCUSED INTERVENTIONS**

**Scapula Mobilization Techniques:**

The patient is positioned in the prone lying position. The therapist places one hand over the acromion process and the other hand over the inferior angle of the scapula. The therapist then moves the scapula through elevation, depression, upward rotation, and downward rotation. Mobilization was applied for 30 seconds in each direction.


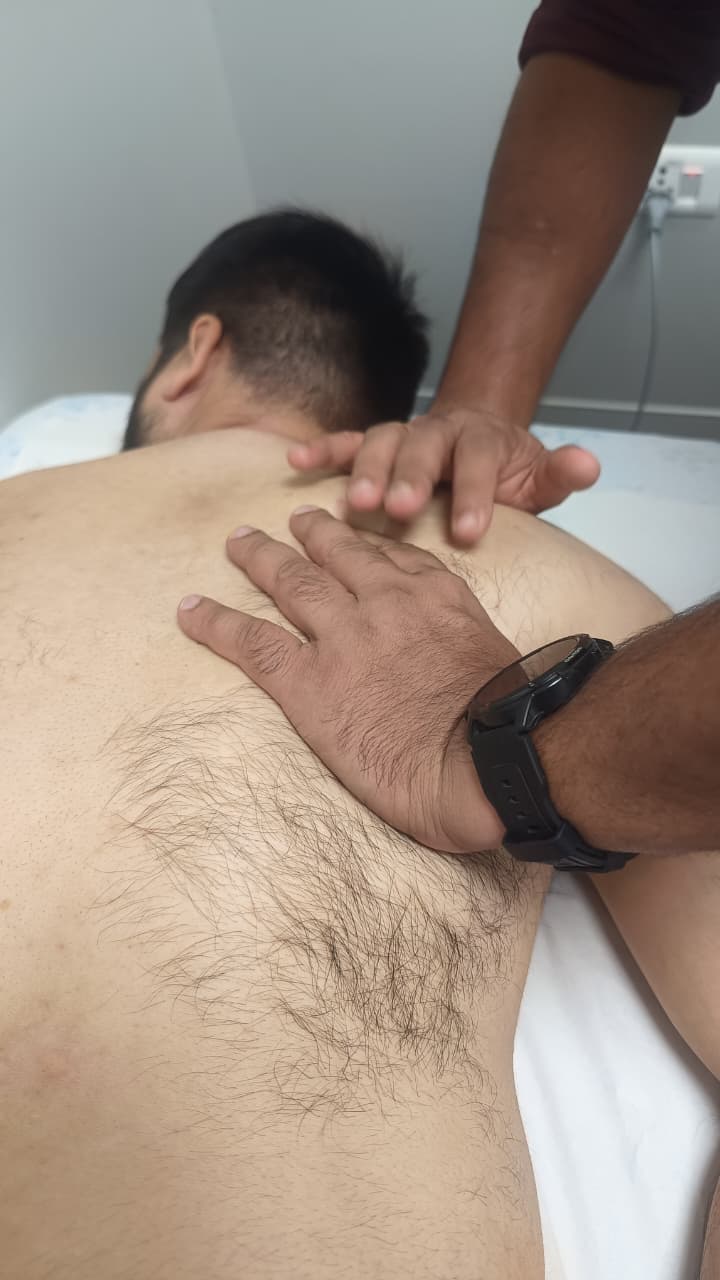


Perform neck and shoulder movements in all planes:

- 2 sets of 10 repetitions

Exercise Protocol: 3 sets, 15 repetitions, 1 minute rest between sets

| Dynamic Hug: Stand with knees slightly bent and feet shoulder-width apart. Begin with elbows flexed to approximately 45°, shoulders abducted to 60°, and internally rotated to 45°. Horizontally flex the shoulders, bringing the arms forward until maximal scapular protraction is achieved. | **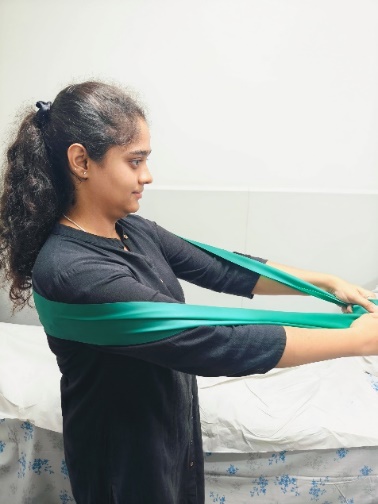** |
| --- | --- |
| Prone Abduction with External Rotation: Lie prone with the shoulder positioned at 90° of forward flexion. Perform horizontal abduction to shoulder level, adding external rotation at the end of the movement. | **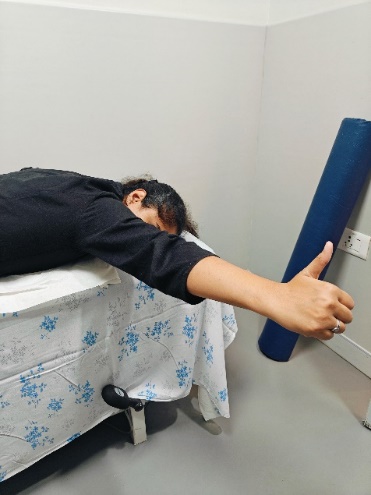** |
| Bilateral External Rotation:  Sit or stand with feet shoulder-width apart and arms at the sides. Hold an exercise band and perform shoulder external rotation. Emphasize posterior tilting of the scapulae. | **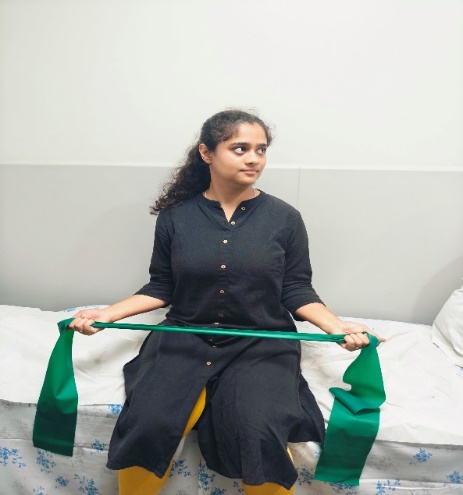** |
| Prone Flexion:  In a prone position, raise the arm overhead in alignment with the lower trapezius muscle fibers. | **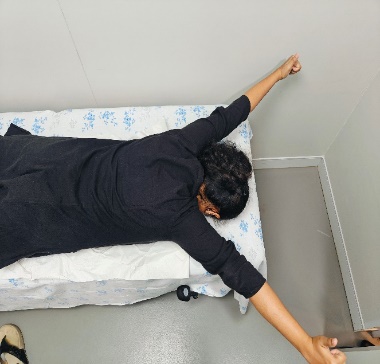** |
| Side Lying External Rotation:  Lie on your side with the shoulder in a neutral position and the elbow flexed to 90°. Place a towel between the trunk and elbow, then perform shoulder external rotation. | **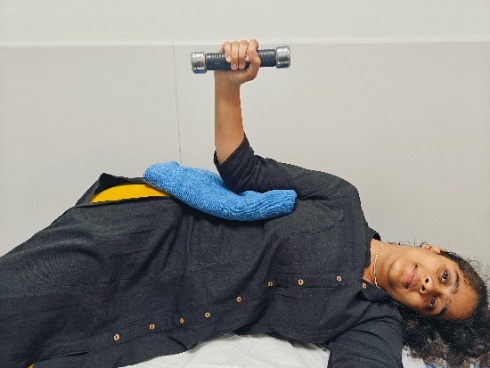** |
| Prone Unilateral Row:  Lie prone with the shoulder at 90° of forward flexion. Extend the shoulder to a neutral position while flexing the elbow to 90°. |  |
| Bilateral Serratus Anterior Punch:  Start with hands at your sides. Extend the elbows and raise the arms to approximately 120° of elevation while fully protracting the scapulae. | **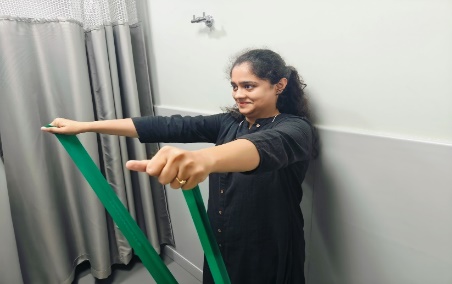** |
| Side Lying Flexion:  In a side-lying position with the shoulder neutral, perform forward flexion in the horizontal plane to approximately 135°. | **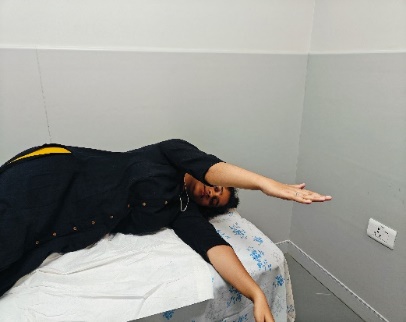** |
| Prone Lying Extension:  Lie prone with the shoulders at 90° of forward flexion. Extend the shoulders back to a neutral position while maintaining neutral rotation. | **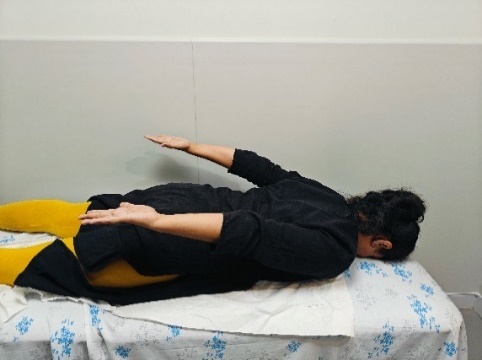** |
| Push-up Plus: Begin in a standard push-up position (or modified on knees or against a surface). Keep the body in a straight line. Protract the scapulae by pushing the upper back toward the ceiling, hold for 2 seconds, then relax and retract the shoulder blades. | **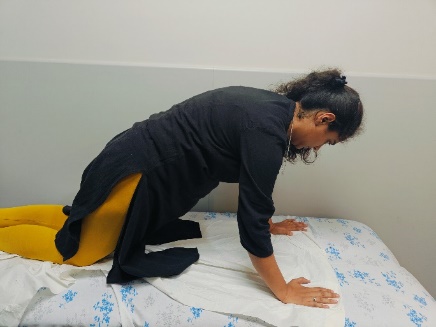** |
| Diagonal Exercise:  Start with the shoulder abducted to 90° and externally rotated in the coronal plane. Perform simultaneous internal rotation and horizontal adduction. |  |
| Upper Trapezius Stretch Sit and hold onto a chair with the hand on the affected side to stabilize the scapula. Rotate the head away from the tight side and use the opposite hand to gently apply an additional stretch. |  |
| Levator Scapulae Stretch: Sit with the head flexed and rotated away from the affected side. Stabilize the scapula by holding the chair. Use the opposite hand to apply gentle overpressure in the direction opposite the muscle pull. | 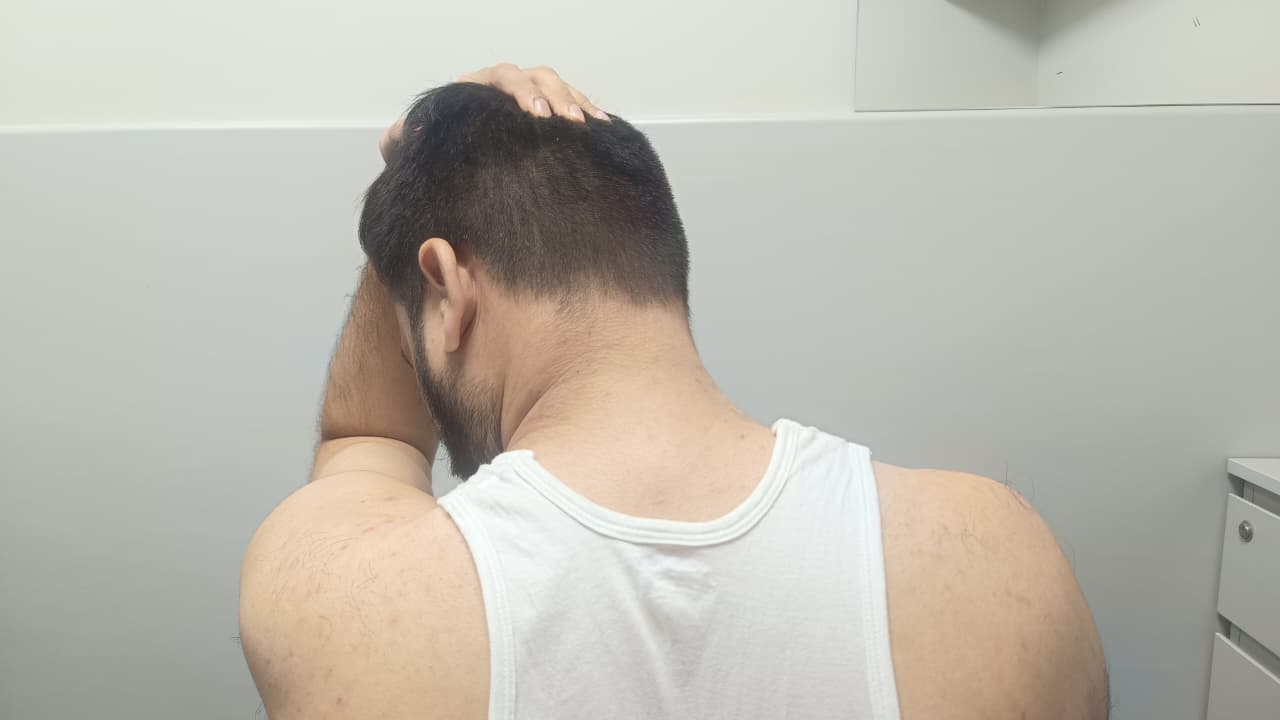 |

**NECK EXERCISES**

**Warm Up**

Active Range of Motion (AROM):
Perform neck movements in all planes:

- 2 sets of 10 repetitions

**Posterior–Anterior (PA) Mobilization of the Spine:**
The therapist stands in front of the patient and applies symmetrical pressure over the transverse processes of the cervical spine. Rhythmic pressure is delivered with a gradual increase in the amount of body weight transmitted through the hands. Mobilization is applied for 30 seconds at each targeted level.

Exercise Protocol: 3 sets, 15 repetitions, 1 minute rest between sets

| Chin Tuck:  In standing, retract the chin (as if making a double chin) while keeping the eyes level. | 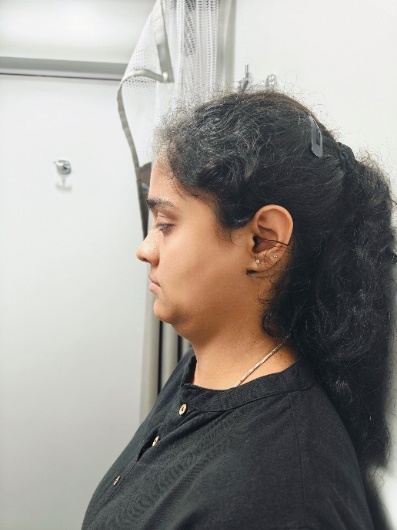 |
| --- | --- |
| Cervical Extension:  In standing, place both hands at the base of the neck and gently extend the neck as far as comfortable. | 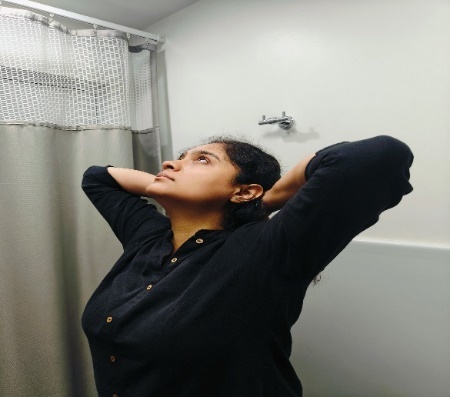 |
| Standing Shrug:  Lift the shoulders upward toward the ears, then relax. | 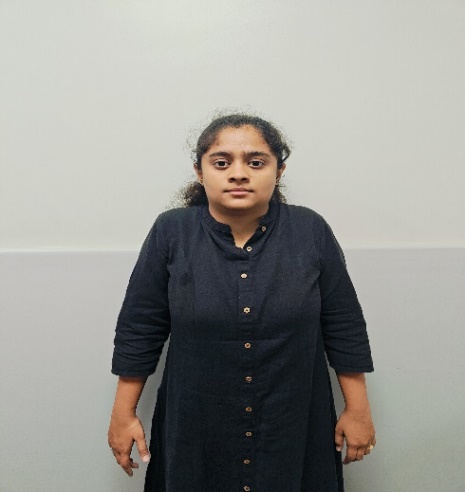 |
| Shoulder Roll:  Roll the shoulders forward in a circular motion, then repeat in a backward direction. | 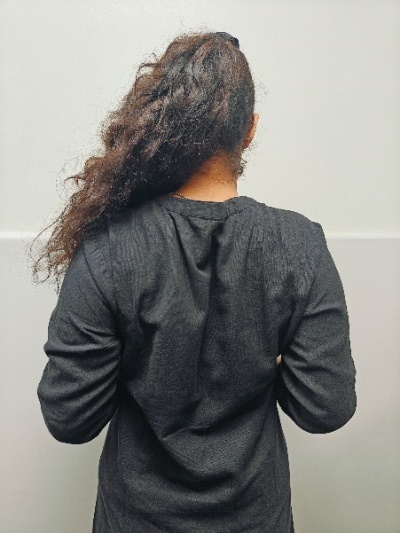 |
| Shoulder Retraction:  Squeeze the shoulder blades together. |  |
| Neck Endurance Exercise:  Sit upright with a resistance band looped around the head and held with the hands. Extend the cervical spine, hold briefly, then slowly return to the starting position while maintaining proper posture. | 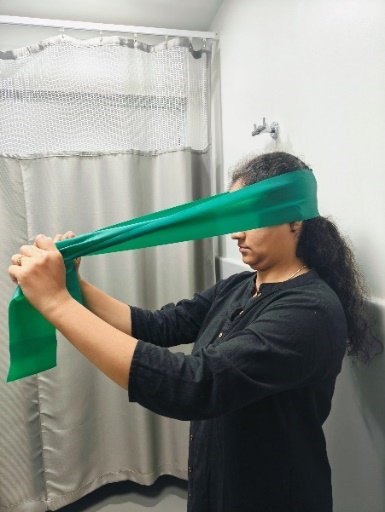 |
| Cervical Protraction:  Sit upright with a resistance band around the head. Flex (protract) the cervical spine against resistance, then slowly return to neutral while maintaining good posture. | 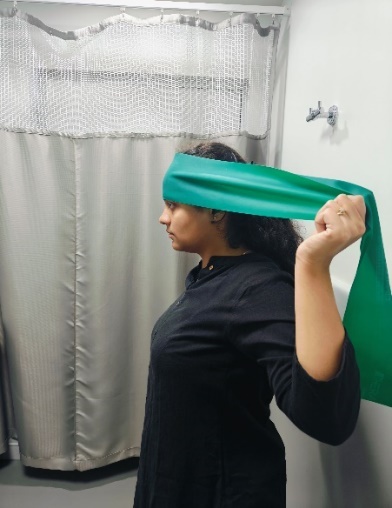 |
| Craniocervical Flexion:  Lie supine with the head in a neutral position. Gently tuck the chin. | 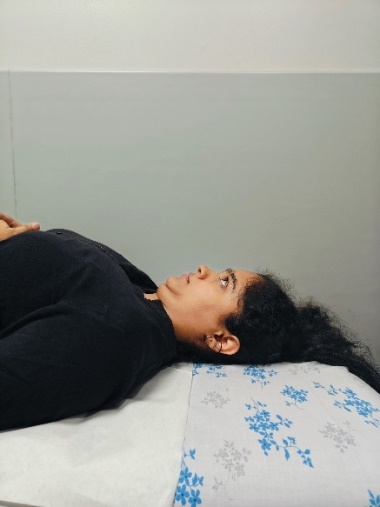 |
| Craniocervical Extension:  Lie prone while maintaining slight craniocervical flexion. Lift the head and neck against gravity and hold. | 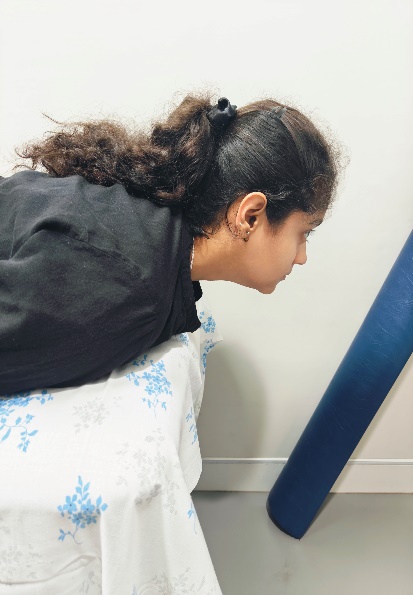 |
